# The Role of Cyclin-Dependent Kinases (CDK) 4/6 in the Ovarian Tissue and the Possible Effects of Their Exogenous Inhibition

**DOI:** 10.3390/cancers15204923

**Published:** 2023-10-10

**Authors:** Graziana Scavone, Silvia Ottonello, Eva Blondeaux, Luca Arecco, Paola Scaruffi, Sara Stigliani, Barbara Cardinali, Roberto Borea, Michele Paudice, Valerio G. Vellone, Margherita Condorelli, Isabelle Demeestere, Matteo Lambertini

**Affiliations:** 1 Department of Medical Oncology, U.O.C. Clinica di Oncologia Medica, IRCCS Ospedale Policlinico San Martino, 16132 Genova, Italy; 2 U.O. Epidemiologia Clinica, IRCCS Ospedale Policlinico San Martino, 16132 Genova, Italy; 3 Department of Internal Medicine and Medical Specialties (DiMI), School of Medicine, University of Genova, 16132 Genova, Italy; 4 S.S. Fisiopatologia della Riproduzione Umana, IRCCS Ospedale Policlinico San Martino, 16132 Genova, Italy; 5 Department of Integrated Diagnostic and Surgical Sciences (DISC), IRCCS Ospedale Policlinico San Martino, 16132 Genova, Italy; 6 Department of Pathological Anatomy, IRCCS Ospedale Gaslini, 16132 Genova, Italy; 7 Research Laboratory on Human Reproduction, Université Libre de Bruxelles, 1050 Brussels, Belgium; 8 Fertility Clinic, Department of Obstetrics and Gynecology, H.U.B—Erasme Hospital, Université Libre de Bruxelles, 1050 Brussels, Belgium

**Keywords:** CDK 4/6 inhibitors, cyclin D-CDK 4/6 complex, gonadotoxicity, ovary

## Abstract

**Simple Summary:**

CDK4/6 inhibitors combined with endocrine therapy are currently the standard of care for the treatment of patients with hormone-receptor-positive (HR+)/Human Epidermal Growth Factor Receptor 2-negative (HER2−) advanced breast cancer and those with high-risk early disease. Oncofertility counseling is a crucial component of the care of young women with newly diagnosed cancer. While the gonadotoxicity of chemotherapy is well established, little is known about the impact of newer targeted agents on ovarian function and fertility. This review aims to clarify the role of the CDK4/6 complex in ovarian tissue by summarizing the preclinical and clinical data available on this topic in order to provide some preliminary guidance on how to counsel women receiving CDK4/6 inhibitors on their potential gonadotoxicity. Dedicated research efforts are needed to improve our understanding of this important issue and perform better oncofertility counseling for young women candidates selected to receive endocrine therapy with a CDK4/6 inhibitor.

**Abstract:**

The combination of cyclin-dependent kinase (CDK) 4/6 inhibitors with endocrine therapy is the standard treatment for patients with HR+/HER2− advanced breast cancer. Recently, this combination has also entered the early setting as an adjuvant treatment in patients with HR+/HER2− disease at a high risk of disease recurrence following (neo)adjuvant chemotherapy. Despite their current use in clinical practice, limited data on the potential gonadotoxicity of CDK4/6 inhibitors are available. Hence, fully informed treatment decision making by premenopausal patients concerned about the potential development of premature ovarian insufficiency and infertility with the proposed therapy remains difficult. The cell cycle progression of granulosa and cumulus cells is a critical process for ovarian function, especially for ensuring proper follicular growth and acquiring competence. Due to the pharmacological properties of CDK4/6 inhibitors, there could be a potentially negative impact on ovarian function and fertility in women of reproductive age. This review aims to summarize the role of the cyclin D-CDK4 and CDK6 complexes in the ovary and the potential impact of CDK4/6 inhibition on its physiological processes.

## 1. Introduction

Breast cancer is the most common malignancy diagnosed among women of reproductive age [1]. The treatment of young breast cancer patients is challenging, since it is associated with additional possible age-related issues [2]. Among the potential negative consequences of anticancer therapies, the risk of developing premature ovarian insufficiency (POI) represents one of the main sources of distress in this patient population [3]. POI might result in long-term sequelae with an impaired quality of life (QoL).

Indeed, several studies have demonstrated the risks and mechanisms of chemotherapy-induced ovarian damage. Chemotherapy agents can induce both structural and functional damage [4]; there is evidence of follicle destruction, characterized by the apoptotic death of oocyte and somatic cells [5,6], as well as vascular damage [7,8] and the premature activation and atresia of primordial follicles [9]. However, limited data are currently available on the use of other newer anticancer therapies including targeted agents.

CDK4/6 inhibitors combined with endocrine therapy are the standard of care for the treatment of patients with HR+/HER2− advanced breast cancer [10]. Four randomized clinical trials have also investigated the role of adjuvant CDK4/6 inhibitors in addition to endocrine therapy in patients with intermediate and high-risk HR+/HER2− early breast cancer. These studies differed for the type of CDK4/6 inhibitors used and for the duration of treatment. Two trials failed to demonstrate improved outcomes for patients receiving adjuvant palbociclib in the PALLAS and penelopeB trials [11,12]. On the other hand, the MonarchE study showed a significant improvement in invasive disease-free survival (iDFS) and distant relapse-free survival (DRFS) at 4 years with the addition of 2 years of adjuvant abemaciclib to endocrine therapy [13]. At a median follow-up of 42 months, the addition of abemaciclib to adjuvant endocrine therapy led to a 6.4% and 5.9% absolute improvement in the 4-year iDFS (hazard ratio (HR) 0.664; 95% confidence interval (CI) 0.578–0.762) and DRFS (HR 0.659; 95% CI 0.567–0.767), respectively [13]. More recently, the NATALEE trial investigating 3 years of adjuvant ribociclib in addition to endocrine therapy reported positive results, albeit after a short follow-up [14]. At a median follow-up of 27.7 months, the addition of ribociclib to adjuvant endocrine therapy led to 3.3% and 2.2% absolute improvements in the 3-year iDFS (HR 0.748; 95% CI 0.618–0.906) and DRFS (HR 0.739; 95% CI 0.603–0.905), respectively [14]. As of now, the only CDK 4/6 inhibitor currently approved for clinical use in the early setting is abemaciclib for patients with high-risk HR+/HER2− early breast cancer.

Investigating the potential gonadotoxicity of CDK 4/6 inhibitors is particularly relevant, considering that almost all patient candidates for this treatment in the curative setting have received prior chemotherapy, with its known impact on ovarian function and reserve. Nevertheless, among the adjuvant trials investigating the addition of a CDK 4/6 inhibitor to endocrine therapy, the phase III penelopeB trial (using palbociclib) is the only one thus far to report the potential gonadotoxicity of this agent [12,15]. The results from the biomarker analysis of this study showed that palbociclib did not significantly affect estradiol and follicle-stimulating hormone (FSH) levels when added to endocrine therapy after chemotherapy. Due to these limited data and the lack of evidence on abemaciclib, fully informed treatment decision making by premenopausal patients concerned about the potential development of POI and infertility and candidates selected to receive this treatment following chemotherapy remains difficult. This review aims to summarize the available evidence on the physiological role of cyclin D and CDK4/6 in the ovarian cell cycle and follicle maturation, as well as data on cell lines and experimental animal models regarding the effect of CDK 4/6 inhibition on ovarian physiological processes.

## 2. Cyclin D-CDK4/6 Complexes

Cell cycle progression from a state of quiescence (G0) to the G1 phase is mediated by cyclin-dependent kinases (CDKs). CDKs are serine/threonine protein kinases that are activated by binding to cyclins and by blocking their inhibitor proteins [16]. CDK4 and CDK6 are considered as promoters of G1 cell cycle progression.

**Figure 1:**
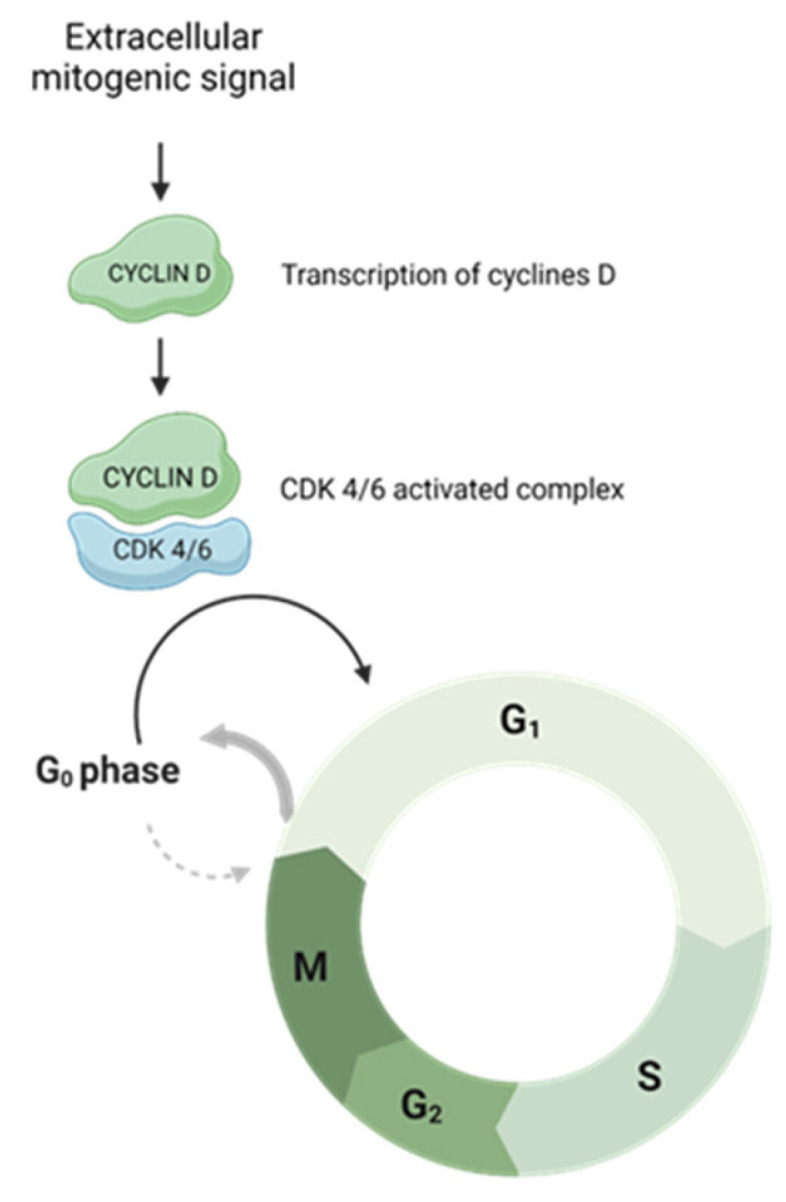
CDK 4/6 complex activation allows entry into G1 phase and cell cycle progression. Created by the authors using BioRender.com, accessed on 22 April 2023.

The activation of specific cyclin–CDK complexes leads to exit from a state of quiescence (G0) and entry into the cell cycle in phase G1 and then to continue through the subsequent steps. Complexes responsible for G0–G1 transition are constituted by the D type cyclins (D1, D2 and D3), CDK4 and CDK6. Once a mitogen signal activates these complexes, the cells are enabled to exit from the state of quiescence and proceed through the G1 phase [17,18]. The cell cycle progression through CDK4 and CDK6 activation is illustrated in Figure 1.

Upon an extracellular mitogenic signal, the cyclin D-CDK4/6 complex is active. This drives the cell cycle towards G0–G1 transition.

D-type cyclins differ from other cyclins in their activation mechanism. While all cyclins are transcriptionally induced by other cyclins during cell cycle progression, the D-type is controlled by an extracellular mitogenic environment. Therefore, they are considered ‘connectors’ between the extracellular microenvironment and the cell cycle machinery [19].

**Figure 2:**
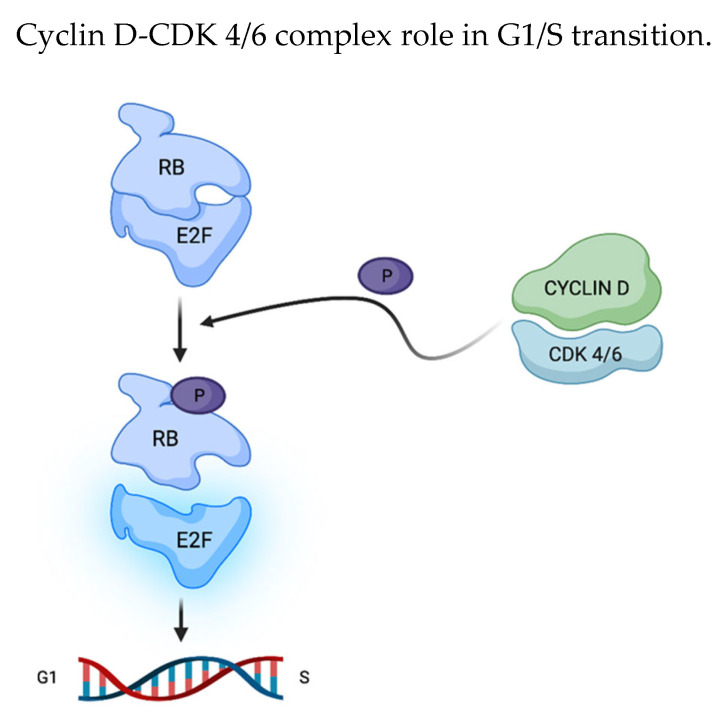
RB phosphorylation leads to the transcription of pro-proliferative genes. Created by the authors using BioRender.com, accessed on 22 April 2023.

The cyclin D-CDK4/6 complex plays a pivotal role in governing the G1/S transition through the retinoblastoma (Rb)-E2F pathway. During the G1 phase, this complex phosphorylates and inactivates Rb. Subsequently, cyclin E-CDK2 drives the phosphorylation process to completion, activating E2F transcription factors to enable the transcription of pro-proliferative genes [20] (Figure 2).

Cyclin D-CDK4/6 complex phosphorylation on RB (retinoblastoma factor)/E2F (transcription factor) is the key event for cell cycle progression: RB phosphorylation enables E2F release and the transcription of pro-proliferative genes.

**Figure 3:**
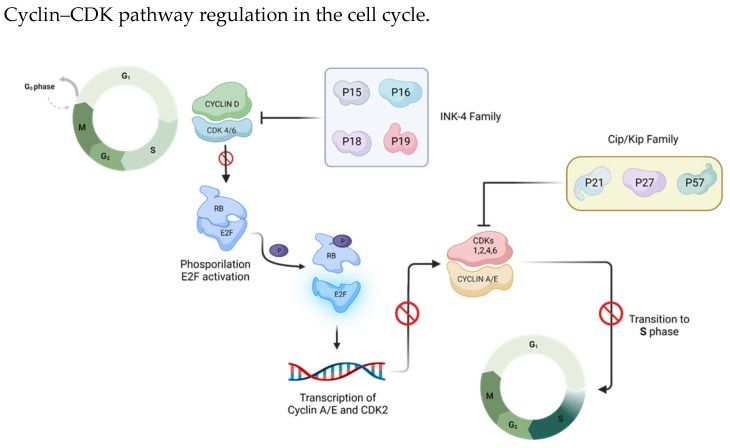
The INK-4 family and the CIP/KIP-type family inhibit the cyclin D-CDK4/6 complex and cyclin A/E-CDK1, CDK2, CDK4 and CDK6, respectively. On the one hand, these complexes’ inactivation leads to a state of cell quiescence in the G0 phase, and on the other, it prevents transition to the S phase. Created by the authors using BioRender.com, accessed on 22 April 2023.

Given their important role in the cell cycle, CDK–cyclin complexes’ interaction is modulated by members of two groups of CDK inhibitors: the CIP/KIP-type family (p21, p27, and p57), which binds to both the cyclin and CDK subunits to inhibit CDK1, CDK2, CDK4 and CDK6 [21], and the INK-type family (p16^INK4a^, p15^INK4b^, p18^INK4c^ and p19^INK4d^), which specifically binds to CDK4 and CDK6 monomers, weakening CDK interaction with cyclin D [22] (Figure 3). In the quiescent state G0, CDK4 and CDK6 are inactive, being bound to the inhibitor INK4, and the expression of cyclin D is absent. As a consequence, Rb is active and represses the transcription factor E2F [18]. After the removal of the repressive proteins INK4, the cells immediately enter the G1 phase and proceed through it through the action of cyclin D, leading to transcriptional activation [23] (Figure 3).

The INK4 family (p16^INK4a^, p15^INK4b^, p18^INK4c^ and p19^INK4d^) and the Cip/Kip family (p21, p27, and p57) are two types of cell cycle endogenous inhibitors. The INK 4 family specifically binds and inhibits cyclin D/CDK 4/6 complexes, whereas the Cip/Kip family can bind both cyclin and CDK subunits to inhibit CDK1, CDK2, CDK4 and CDK6.

## 3. Cyclin D-CDK 4/6 in Ovarian Development

CDK4 and CDK6, as well the three D-type cyclins, are essential for proliferation in several cell types [24]. Mouse embryos defective for either CDK4 or CDK6 display normal organogenesis [25] but die during the late stages of embryonic development due to severe anemia [26]. This suggests that, at least in mice, each of these CDKs can compensate for the ablation of the other and thus be compatible with life, whereas the loss of both kinases primarily affects the proliferation of erythroid progenitors.

This behavior may be explained by the fact that embryo cells have a short G1 phase and do not stop at the G0 stage, and they do not require all the cellular mechanisms involved in the signaling of cell cycle re-entry from G0 [27].

On the other hand, in adult differentiated tissues, studies reported ovarian failure related to CDK4 deficiency. CDK4-deficient mice have small ovaries, defects in corpus luteum formation and a reduced ovulation efficiency, as well as embryo implantation failures [28]. CDK4-deficient mice were shown to be sterile; they are characterized by extended estrus cycles and reduced serum progesterone levels [29,30]. A subsequent investigation revealed that administering daily progesterone injections successfully reinstated implantation and fertility in CDK4-deficient mice [31]. These findings strongly suggest that infertility in mice lacking CDK4 is likely associated with diminished serum progesterone levels. However, the complete mechanism underlying this infertility is not yet fully understood.

The limited manifestations of CDK4 deficiency could be explained by the ability of CDK6 to replace the function of CDK4 [19]. On the other hand, CDK6 deficient mice are not sterile [25].

These results indicate that cyclin-dependent kinases such as D-type cyclin-dependent kinases are not essential for cell cycle entry and suggest the existence of alternative mechanisms to initiate cell proliferation upon mitogenic stimulation. In addition, these findings indicate that CDK4 and CDK6 could have overlapping functions.

## 4. Cyclin D in Granulosa Cells

In the ovary, granulosa cells are the somatic cells responsible for ovarian function. Granulosa cell cycle progression is crucial for follicular maturation, ovulation, and the genesis of the corpus luteum [24,26].

Inside primordial follicles, granulosa cells are suspended in the G0 phase. However, upon follicular activation, these cells re-enter the cell cycle. In rodents, this event leads to the substantial development of preovulatory follicles within 3 days [27] (p. 20).

In rats, sheep [32] and humans [33], the proliferation rates of granulosa cells change with the stage of maturation of the follicle and its size: cell cycle progression is slow when follicles are recruited from the primary to secondary stage of development, while the granulosa cells of the preantral follicles initiate an increase in the rate of cycle progression.

In this context, FSH is crucial for promoting granulosa cell proliferation through the up-regulation of cyclin D2, while luteinizing hormone (LH), via MAPK1/3 activation, causes cell cycle arrest by activating CDK endogenous inhibitors [34].

The functions of cyclin D1, D2, and D3 appear to be specific to different organs. Cyclin D2 plays a particularly predominant role in the development and function of the gonads [35], and it is specifically expressed in granulosa cells. Its expression increases under FSH, estradiol and insulin stimuli [36]. FSH stimulation, in fact, results in the formation and subsequent activation of the cyclin D2/CDK4 complex, responsible for the initiation of DNA synthesis [37]. On the other hand, LH, via MAPK1/3 activation, causes cell cycle arrest by activating CDK endogenous inhibitors [38].

In the cultured granulosa cells of rats, it has been shown that the expression of cyclin D2 is FSH-dependent and that this molecule has a crucial role in the growth and maturation of follicles [32,33].

Consistent with this, female mice deficient in cyclin D2 display a mutant-like ovarian phenotype, with reduced granulosa cell proliferation, anovulation and infertility [27,32].

**Figure 4:**
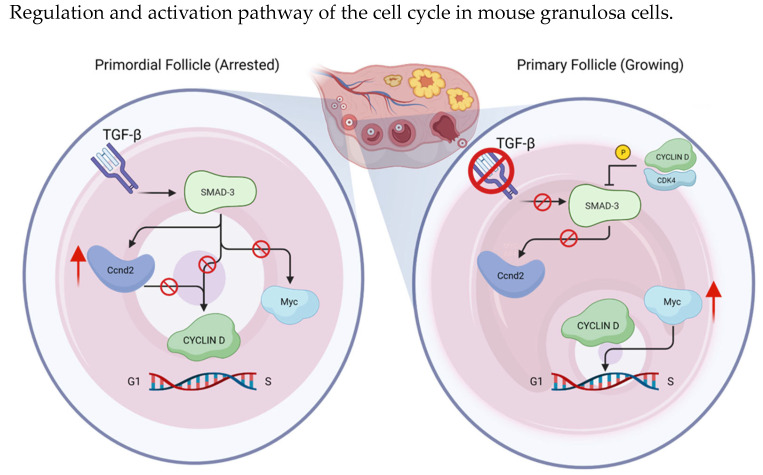
Primordial follicles are in a quiescence state as a result of SMAD3 activation by TGFβ factor. However, when SMAD3 is inactive, progression in growing follicles is enabled. Created by the authors using BioRender.com, accessed on 22 April 2023.

Another regulatory factor of cyclin D2 is SMAD3, whose transcription is activated by Transforming Growth Factor Beta (TGFβ); it binds the cyclin D2 promoter, maintaining inhibited transcription [30]. TGFβ-mediated SMAD2/3 transcription factors are specifically expressed in primordial granulosa cell nuclei, and TGFβ signaling is known to be important for maintaining growth arrest in various cell types. Evidence suggests that cyclin D-CDK4 complex phosphorylates and inhibits SMAD3 [19]. Furthermore, it seems to directly bind the Ccnd2 and Myc genes, regulating their transcription. In arrested primordial follicles, SMAD3 promotes Ccnd2 expression and represses Myc, ensuring that granulosa cells do not progress in the cell cycle. The eventual loss of SMAD3 leads to the transcription of Myc and progression in growing follicles [30] (Figure 4).

SMAD-3 phosphorylation and its resulting inhibition by the cyclin D/CDK4 complex is crucial to G1-S transition in cell cycle progression; SMAD3 activation by TGFβ promotes Ccnd2 (Cyclin D2) expression and represses Myc, ensuring that granulosa cells do not progress in the cell cycle, keeping the follicle in a primordial condition.

## 5. Cyclin D-CDK4/6 Complexes in the Oocytes

To reach the state of full competence, oocytes undergo several morphological and molecular modifications. Around birth, oocytes are arrested in the meiotic prophase I, and they remain in this stage until puberty, when the preovulatory surge of LH causes ovulation. After the LH surge, oocytes pass through the G2/M transition, enter anaphase, and then complete the first meiosis. Meiotic oocytes achieve their maturation in the metaphase of meiosis II without entering the S phase and are arrested in this stage until fertilization.

Several molecules are involved in regulating meiosis resumption in oocytes. Prophase I arrest results from the maintenance of low activity of the complex cyclin B1-CDK1, known as maturation promoting factor (MPF), through the phosphorylation-mediated inactivation of CDK1 and degradation of cyclin B1. The LH surge activates CDK1, which both phosphorylates different meiotic phosphoproteins and protects them from dephosphorylation by inhibiting the activities of CDK1-antagonizing protein phosphatases [39,40]. Although there is no S phase in meiosis, the cell cycle regulators of G1/S transition during mitosis may be involved in meiotic progression. Regarding CDK4/6, the cyclin D-CDK4/6 complex and p27—the CDK4 inhibitor—were found to be expressed in mouse oocytes [41]. The dynamics of their amounts, structure and localization strongly suggest their involvement in the regulation of oocyte development, but whether the cyclin D-CDK4/6 complex regulates meiotic cell cycle progression is not known. More recently, Dong et al. [42] observed different roles of the cyclin D-CDK4/6 complex in oocyte meiosis.

CDK4/6 inhibition can lead to the dysfunction of the main phases of meiosis. In particular, it can interfere with spindle assembly checkpoint (SAC) activity, leading to aberration in chromosome segregation; this results in an incorrect number of chromosomes (aneuploidy) in the oocyte. The inactivation of SAC causes a premature loss of cyclin B1 via ubiquitin-mediated degradation; moreover, there is a decrease in MPF activity in meiotic oocytes.

Therefore, the cyclin D-CDK4/6 complex is required for regulating meiotic progression in mouse oocytes and mediating control of the SAC to prevent aneuploidy in female meiosis I.

## 6. Role of Exogenous CDK Inhibitors (CDKi) in the Ovary

Limited data have been published about the role of exogenous CDKi in the ovary and their possible gonadotoxicity.

The specific CDK4/6 inhibitor palbociclib has a role not only in CDK4/6 activity but also in activation through the phosphorylation of the bulk of cyclin D-CDK4 complexes. A study conducted on Chinese hamster ovarian cells (CHO) showed that, in absence of the inhibitor p21, palbociclib treatment increased the stability and activation of cyclin D3-CDK4/6 complexes [43]. This effect was not observed in the presence of p21 co-expression, suggesting that palbociclib and p21 competed for the stabilization of cyclin D3-CDK4 complexes. Furthermore, recent studies [44,45] showed a potential “protective” role of palbociclib in granulosa cells in regard to chemerin, which is an adipokine secreted mainly from adipose tissue that causes reactive oxygen species (ROS) accumulation and triggers apoptosis in mouse granulosa cells. Evidence suggests that palbociclib has an antiapoptotic effect on immortalized human granulosa-lutein (hGL) cells treated with chemerin. These results can be explained by the fact that palbociclib can diminish ROS generation in hGL cells and suppresses chemerin-induced apoptotic protein expression.

Nevertheless, another study showed that CDK4/6 inhibitor activity causes incomplete DNA replication, leading to cell damage. This drives the cell to exit the cell cycle in a p53-dependent manner [46]. Indeed, if cells fail to withdraw from the cell cycle following DNA replication problems, they enter mitosis with fragmented chromosomes and excessive DNA damage, which further limits their proliferative potential, leading to senescence [46].

In fact, the data obtained in this study using a retinal epithelial cell (RPE1) line demonstrated that G1 arrest becomes problematic in long-term treatment [46].

A comparison between immortalized RPE1 and a type of breast cancer cell (MCF7) was performed in order to reveal potential differences in cellular response to CDK4/6 inhibition. Following treatment with palbociclib, both breast cancer and RPE1 cells underwent DNA damage, but its consequences were worse in healthy cells. Indeed, 3 days of exposure to palbociclib treatment were needed to reduce the long-term proliferation of RPE1 cells to a level comparable to that reached after 7 days of therapy in breast cancer cells. Hence, the study links CDK4/6 inhibitors to genotoxic stress, which can occur in healthy cells, such as RPE1 and granulosa cells [46]. CDK4/6 inhibitors induce cell cycle arrest in tumor cells, leading to apoptosis [47,48]. As recently demonstrated in the 2022 study of Crozier et al., this apoptotic effect can also be seen in healthy cells (RPE1).

Overall, the data on palbociclib are conflicting and cannot yet confirm a potential damaging role of CDK4/6 exogenous inhibition in the ovary and specifically in granulosa cells.

To date, there are no consistent data on abemaciclib and ribociclib ovarian toxicity.

## 7. Conclusions

Cyclins and their associated CDKs are key regulators of cell cycles, contributing to the transition from the G1 to S phase [49]. Considering their crucial role in controlling proliferation, senescence, migration, apoptosis and angiogenesis, the dysregulation of cyclin–CDK represents a hallmark of cancer.

In the ovary and, more specifically, in the granulosa cells of primordial follicles, the consecutive activation of different CDKs leads the cell to move from a quiescent state in the G0 phase to the G1 phase, back into the cell cycle. The transition into these different phases is regulated by the activation of specific cyclin–CDK complexes. Following a mitogenic signal, the D type of cyclins and CDK 4 and 6 are the first complexes to be activated. Once this checkpoint is bypassed, the cell proceeds through the G1 phase and commits to the completion of the mitotic cycle and divide.

The impact of the currently available CDK4/6 inhibitors ribociclib, abemaciclib and palbociclib on healthy ovarian cells has not yet been fully investigated. In this regard, the currently available data are limited and controversial. On the one hand, research has shown a possible protective role of palbociclib in ovarian cells; on the other hand, it appears that this agent could lead healthy cells to a state of replication stress due to cell cycle interruption resulting in excessive DNA damage, which further limits their proliferative potential.

The growing use of CDK4/6 inhibitors in the clinical setting and the paucity of data on their potential gonadotoxicity highlights the need to pursue dedicated research efforts in this area to further improve treatment decision making for premenopausal women who are candidates for this targeted treatment.
